# Urinary 8-oxo-7,8-dihydroguanosine as a Potential Biomarker of Aging

**DOI:** 10.3389/fnagi.2018.00034

**Published:** 2018-02-27

**Authors:** Wei Gan, Xin-Le Liu, Ting Yu, Yuan-Gao Zou, Ting-Ting Li, Shuang Wang, Jin Deng, Lan-Lan Wang, Jian-Ping Cai

**Affiliations:** ^1^Department of Laboratory Medicine, West China Hospital, Sichuan University, Chengdu, China; ^2^The MOH Key Laboratory of Geriatrics, Beijing Hospital, National Center of Gerontology, Beijing, China

**Keywords:** 8-oxoguanosine, urine, mass spectrometry, method validation, biomarkers of aging

## Abstract

**Background:** A molecular biomarker of physiologic age, as opposed to chronologic age, is needed in clinical medicine. 8-oxo-7,8-dihydro-2′-deoxyguanosine (8-oxodGsn) and 8-oxo-7, 8-dihydroguanosine (8-oxoGsn) are two promising aging biomarkers.

**Methods:** A total of 1,228 healthy Chinese residents (613 males and 615 females) 2–90 years of age were randomly selected. Spot urine samples were collected, and the concentrations of 8-oxodGsn and 8-oxoGsn were measured using ultra-high-performance liquid chromatography with a triple quadrupole mass spectrometer (UPLC-MS/MS). Method validation, including accuracy, precision, linearity and quantification limit, was performed. The relationship between oxidized guanosine and age/gender was evaluated.

**Results:** 8-oxodGsn and 8-oxoGsn were eluted at 1.61 and 1.30 min, respectively. The calibration curve was linear in the range of 0.2–500 ng/ml for both analytes. The lowest limit of quantification (LLOQ) was 0.2 ng/ml for 8-oxodGsn and 0.1 ng/ml for 8-oxoGsn. There was an age-dependent increase in the biomarkers from the 21- to 30-year-old group to the 81- to 90-year-old group in both genders. In the subjects older than 61 years of age, the levels of 8-oxodGsn as well as 8-oxoGsn in urine were much higher in females than in males. The content of 8-oxoGsn correlated more closely with age and was higher (approximately 2-fold) than that of 8-oxodGsn for a given individual.

**Conclusions:** 8-oxodGsn and 8-oxoGsn can be easily measured by UPLC-MS/MS. Urinary 8-oxoGsn may be a potential biomarker to determine a person's physiologic age and identify individuals at high risk of developing age-associated disease.

## Introduction

Aging is a complex process that can be defined as an irreversible decline in the functional capacity and stress resistance associated with increased risk of morbidity and mortality. The rate of aging differs among individuals due to variations in the genetic and environment background. Chronological age, which is simply calculated according to birth date, is an imprecise measure of biological aging. The disconnection between chronological age and lifespan has led to a search for effective and validated biomarkers of aging.

A good aging biomarker should be based on mechanisms described by major theories of aging, which mainly include oxidative stress, protein glycation, DNA methylation, inflammation, cellular senescence and hormonal deregulation (Banerjee et al., 2011; Horvath, 2013; Catera et al., 2016; Sebastiani et al., 2017). The current consensus is that aging is driven by the lifelong gradual accumulation of a broad variety of molecular faults in the cells and tissues. Nucleic acids, as the basic genetic material, play a vital role in the protein synthesis. Any error occurring on a DNA template or in messenger RNA will eventually lead to the production of abnormal proteins. However, the exposure of a double-stranded DNA chain or single-stranded RNA chain to free radicals, including hydroxyl radical (^−^OH), superoxide anion (${\text{O}}_{2}^{-}$) and nitric oxide (NO^−^), which are by-products of normal metabolism, can cause oxidative damage to biomolecules.

8-Oxo-7,8-dihydro-2′-deoxyguanine (8-oxodGsn, also known as its isomer 8-hydroxy-2′-deoxyguanine, 8-OHdGuo) is by far the most studied DNA oxidative product because of its C-8 position's vulnerability to reactive oxygen species (ROS) and its mutagenic potential (Sekiguchi, 2006). If left unrepaired, this lesion can result in a G-to-T transversion mutation (Cheng et al., 1992). Similarly, mismatch of 8-oxo-7,8-dihydroguanine (8-oxoGsn) in RNA to adenine (A) leads to transcriptional errors and produces abnormal protein (Tanaka et al., 2007; Kamiya et al., 2009). Oxidized DNA can be repaired by a series of glycosylases that are specific to particular oxidized bases and possibly by non-specific excision repair enzymes (David et al., 2007). These excision products can be transported across the cell membranes and excreted into cerebrospinal fluid (CSF), plasma and urine without any further metabolism (Cooke et al., 2000).

Among the available measurement methods, enzyme-linked immunosorbent assay (ELISA) has been employed most extensively to determine the amounts of 8-oxodGsn and 8-oxoGsn derived from bodily fluids (Garratt et al., 2010). However, this method overestimates the levels of 8-oxodGsn and 8-oxoGsn because the antibodies used sometimes exhibit cross-contamination of 8-oxodGsn and 8-oxoGsn. Chromatographic methods, including high-performance liquid chromatography-electrochemical detection (HPLC-ECD) (Hofer et al., 2006), liquid chromatography with tandem mass spectrometry (LC-MS/MS) (Weimann et al., 2002) and gas chromatography-mass spectrometry (GC-MS) (Ravanat et al., 1999; Lin et al., 2004), have been developed for obtaining more accurate quantification. Most notably, LC-MS/MS has distinct advantages over other methods. Sample impurities can be separated during the HPLC phase, and all types of guanosines can then be distinguished by tandem mass spectrometry according to their molecular weights, which cannot be separated by conventional HPLC.

We previously established an LC–MS/MS-based system and determined the oxidized nucleosides in senescence-acceleration-resistant mouse 1 (SAMR1), demonstrating that the measurement of 8-oxoGsn in urine had potential as a novel means of evaluating the aging process (Gan et al., 2012). However, some technical conditions remain to be optimized. For example, a whole run for a single urine sample lasted 14 min, which is not suitable for large-population investigation. In addition, the studies were performed using only samples derived from mice.

In the present study, we applied this procedure to human urine samples to see if such samples can be used to estimate the physiologic age.

## Materials and methods

### Participants and samples

Participants were recruited from among those undergoing routine health checkups carried out by their employers in West China Hospital, Sichuan University from January to April, 2016. Most of them were Sichuanese. Those who met the following inclusion criteria were recruited in this study: (Catera et al., 2016) no history of somatic or psychiatric abnormalities registered in their medical records and (Horvath, 2013) no history of medication, smoking or alcohol consumption during the preceding 2 weeks. Some important parameters pertaining to BMI, blood pressure, blood glucose, lipids, liver function and kidney function of the participants were summarized in Supplementary Table 2. Children participants were recruited from West China kindergarten.

The study was approved by the Ethics Committee of West China Hospital, Sichuan University and the approvement number is 2014 Clinical Trial (No.14). Adult participants and parents of the children participants signed an informed consent form after they were informed about the objective of the study.

Spot urine samples were collected, and concentrations of 8-oxodGsn and 8-oxoGsn were analyzed using ultra-high-performance liquid chromatography with a triple quadrupole mass spectrometer (UPLC-MS/MS). Creatinine in urine was measured using a Cobas P800 system (Roche Diagnostics GmbH, Germany) in the Department of Laboratory Medicine, West China Hospital, Sichuan University, which is a College of American Pathologists (CAP)-accredited laboratory. The test is called a “compensated” Jaffe assay, in which a fixed concentration is automatically subtracted from each result to correct for non-specific reactions caused by serum pseudo-creatinine chromogens. Urinary 8-oxodGsn and 8-oxoGsn levels were normalized relative to the amount of creatinine.

### Chemicals

8-Oxo-2′-deoxyguanosine (8-oxodGsn, >98% purity) was purchased from Sigma-Aldrich (USA). 8-oxoguanosine (8-oxoGsn, >98% purity) was purchased from ALEXIS Biochemicals (USA). [^13^C,^15^N_2_]8-oxoGsn was customized from Toronto Research Chemicals (Canada). Formic acid and methanol were of HPLC grade and purchased from Fisher Scientific (USA). The water applied in the determination process was deionized at 18.2 MΩ.

### Instruments

A Waters ACQUITY UPLC system equipped with a Xevo TQ-S triple quadrupole mass spectrometer with an ESI source was used for UPLC-MS/MS. Chromatographic separation was performed on an ACQUITY UPLC system (Waters Corporation, USA) using an ACQUITY UPLC BEH C18 column (1.7 μm, 2.1 × 50 mm; Waters Corp.) and a Van Guard precolumn (1.7 μm, 2.1 × 5 mm; Waters Corp.) with a column temperature of 25°C.

The mobile phase contained 0.1% formic acid (A) and 100% methanol (B), and gradient elution was applied to obtain the best peak shape (Supplementary Table 1). Electrospray ionization was performed in the positive ion mode. The multiple reaction monitoring (MRM) mode was applied during quantification. The desolvation temperature was set at 500°C. Capillary and cone voltages were set at 2.5 KV and 20 V, respectively. Quantification was based on the signal peak area from transitions m/z 283.9 → 167.9 (8-oxodGsn) and 299.9 → 167.9 (8-oxoGsn) related to the peak area of the [^15^${\text{N}}_{2}^{13}$C_1_]8-oxoGsn m/z 302.9 → 170.9.

### Urine sample preparation

The frozen urine was thawed by incubation at 37°C for 5 min. Each sample was separated into two parts. One aliquot was used for the UPLC-MS/MS analysis to quantify 8-oxodGsn and 8-oxoGsn. The other aliquot was used to quantify creatinine to normalize the dilutions of the urine.

For MS/MS measurement, 1 ml of urine was collected, and a 400 μl-aliquot of the supernatant was mixed with 4 μl of 500 ng/ml [^15^${\text{N}}_{2}^{13}$C_1_]8-oxoGsn internal standard and 4 μl of formic acid (FA). The mixture was mixed in a vortex mixer and centrifuged at 12,000 × g for 5 min, and the supernatant was used for analyses.

### Method validation

#### Linearity and sensitivity study

Standard solutions of 0.05, 0.1, 0.2, 0.39, 0.78, 1.56, 3.125, 6.25, 12.5, 25, and 50 ng/ml of 8-oxodGsn and 8-oxoGsn were analyzed. The analysis was performed in triplicate. The quantification limit was defined as a signal-to-noise ratio of 10:1.

#### Accuracy study

Accuracy was determined from the recovery test. A urine sample with 8-oxodGsn (0.22 ng/ml) and 8-oxoGsn (1.56 ng/ml) was chosen as the background urine. Ten microliters of 8-oxodGsn (50 ng/ml) and 8-oxoGsn (100 ng/ml) containing sample were spiked into 1 ml of urine to obtain low-level urine, 10 μl each of a mixture of 8-oxodGsn (500 ng/ml) and 8-oxoGsn (1 μg/ml) were spiked into 1 ml urine to obtain medium-level urine, and 10 μl of 1 μg/ml of 8-oxodGsn and 10 μl of 2 μg/ml of 8-oxoGsn mixture were spiked into 1 ml of urine to obtain high-level urine. The concentrations in the spiked urine samples were detected, and the recovery rates were calculated. The recovery study was performed in triplicate.

#### Within-day and between-day precisions

For within-day precision, urine at 3 levels (low: 0.23 and 1.56 ng/ml, medium: 3.94 and 7.20 ng/ml, high: 12.91 and 26.52 ng/ml for 8-oxodGsn and 8-oxoGsn, respectively) were analyzed consecutively 20 times in a single day. For between-day precision, the same urine samples were analyzed consecutively for 20 days. Precision was expressed as the percentage of the relative standard deviation (RSD, %). The precision study was also performed in triplicate.

### Statistical analyses

All statistical analyses were performed using the SPSS 16.0 software program (SPSS Inc. USA). Urinary 8-oxodGsn and 8-oxoGsn followed a log-normal distribution, whereas creatinine was distributed normally. To make the figure look simpler and clearer, the levels of 8-oxodGsn/crea and 8-oxoGsn/crea were expressed as the mean±standard deviation. Differences between gender groups were assessed by Student's *t*-test. Analyses of the relationship between guanine-related compound levels and age were carried out using linear correlation with Pearson's correlation coefficient. All *p*-values were two-sided, and a *p* < 0.05 was considered statistically significant.

## Results

### UPLC-MS/MS measurement of 8-oxodGsn and 8-oxoGsn in human urine

**Figure 1:**
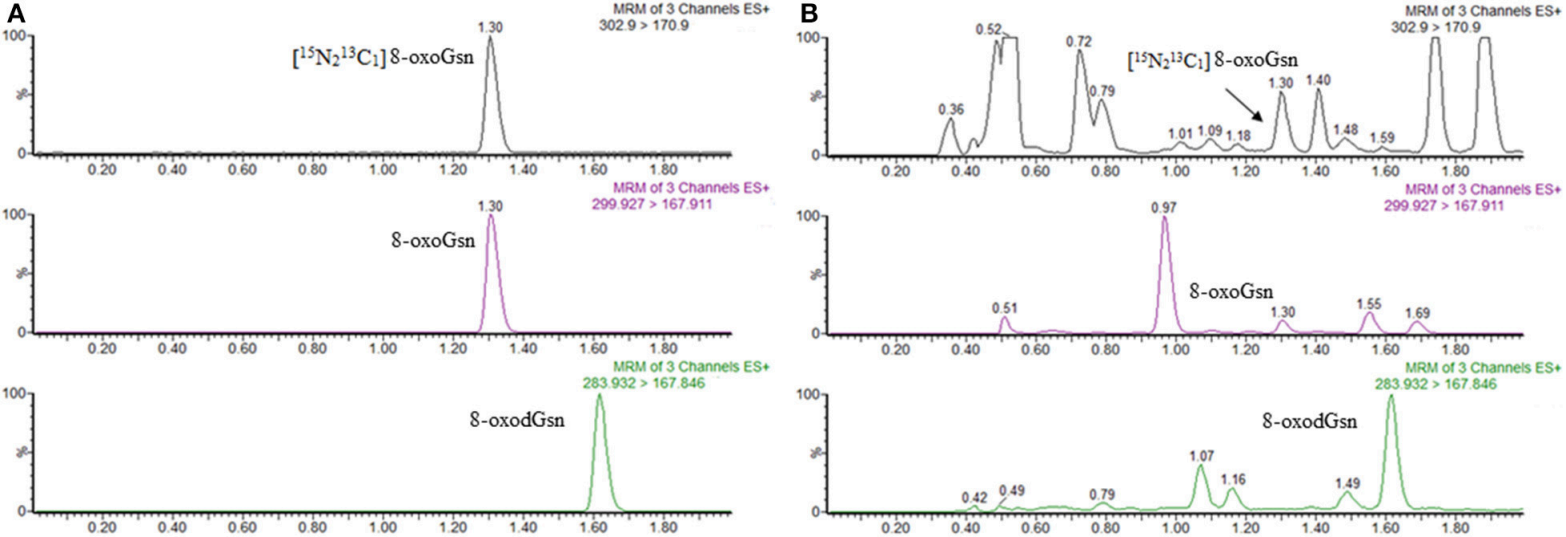
UPLC-MS/MS chromatograms of 8-oxoGsn and 8-oxodGsn. **(A)** Chromatograms of 8-oxoGsn and 8-oxodGsn standard. **(B)** Chromatograms of a urine sample.

A whole run for one sample took 3 min using the present UPLC-MS/MS method, which was much faster than previously reported methods (Weimann et al., 2002; Lin et al., 2004) and our previous method (Gan et al., 2012; Nie et al., 2013). Figure 1 shows the findings of chromatography for 8-oxodGsn and 8-oxoGsn. 8-oxodGsn and 8-oxoGsn were eluted at 1.61 and 1.30 min, respectively, without cross-contamination under the optimized UPLC elution conditions.

The calibration curve was linear in the range of 0.2–500 ng/ml for both analytes. Even 500 ng/ml of 8-oxoGsn did not overload the mass detector. The present UPLC-MS/MS method showed better sensitivity than our previously reported HPLC-MS/MS method. The detection limit of urinary was as low as 0.2 ng/ml (S/N = 9.48) for 8-oxodGsn and 0.1 ng/ml (S/N = 11.59) for 8-oxoGsn when 2 μl of urine was injected. These LLOQs were lower than most of the urinary 8-oxodGsn and 8-oxoGsn levels reported.

After screening spot urine samples from more than 100 people with different ages and diagnoses, we defined urine with 0.23 ng/ml 8-oxodGsn and 1.56 ng/ml 8-oxoGsn as low level, 3.94 ng/ml 8-oxodGsn and 7.20 ng/ml 8-oxoGsn as medium level and 12.91 ng/ml 8-oxodGsn and 26.52 ng/ml 8-oxoGsn as high level. These three levels of urine were used in within-day and between-day precision assays.

The within-day precision was 2.0, 0.68, and 1.31% for 8-oxoGsn and 10.0, 6.43, and 4.92% for 8-oxodGsn in low-, medium- and high-level urine samples, respectively. The between-day precision was 4.96, 2.98, and 2.96% for 8-oxoGsn and 16.87, 10.88, and 8.90% for 8-oxodGsn in low-, medium- and high-level urine samples, respectively. The inter-day repeatability of low-level 8-oxodGsn was not very good (CV = 16.87%) because the concentration of the chosen sample was lower than most of the urine sample.

The recovery was consistently good over a wide range of urine samples (from very dilute to very concentrated). The average recovery was 102.2% for 8-oxoGsn and 95.9% for 8-oxodGsn.

Blank solvent samples analyzed within the batch verified that there was no detectable carry-over from urine samples or the highest standard sample. The oxidative adducts were stable in human urine for at least 5 days at room temperature, which will facilitate sample collection from remote, rural areas.

### Effects of gender on the contents of urinary 8-oxodGsn and 8-oxoGsn

In total, 1,228 Chinese residents (613 males and 615 females, from 2 to 90 years of age) were selected. They were subdivided into 10-year age classes (e.g., 1–10 years, 11–20 years, etc.). The distribution of participants among gender and age classes is shown in Table 1.

**Table 1:** Distribution of participants by gender and age group.

**Age (years)**	**Male**	**Female**	**Total**
1–10	51	51	102
11–20	52	18	70
21–30	79	88	167
31–40	89	97	186
41–50	102	100	202
51–60	102	103	205
61–70	48	67	115
71–80	68	81	149
81–90	22	10	32
Total	613	615	1,228

**Figure 2:**
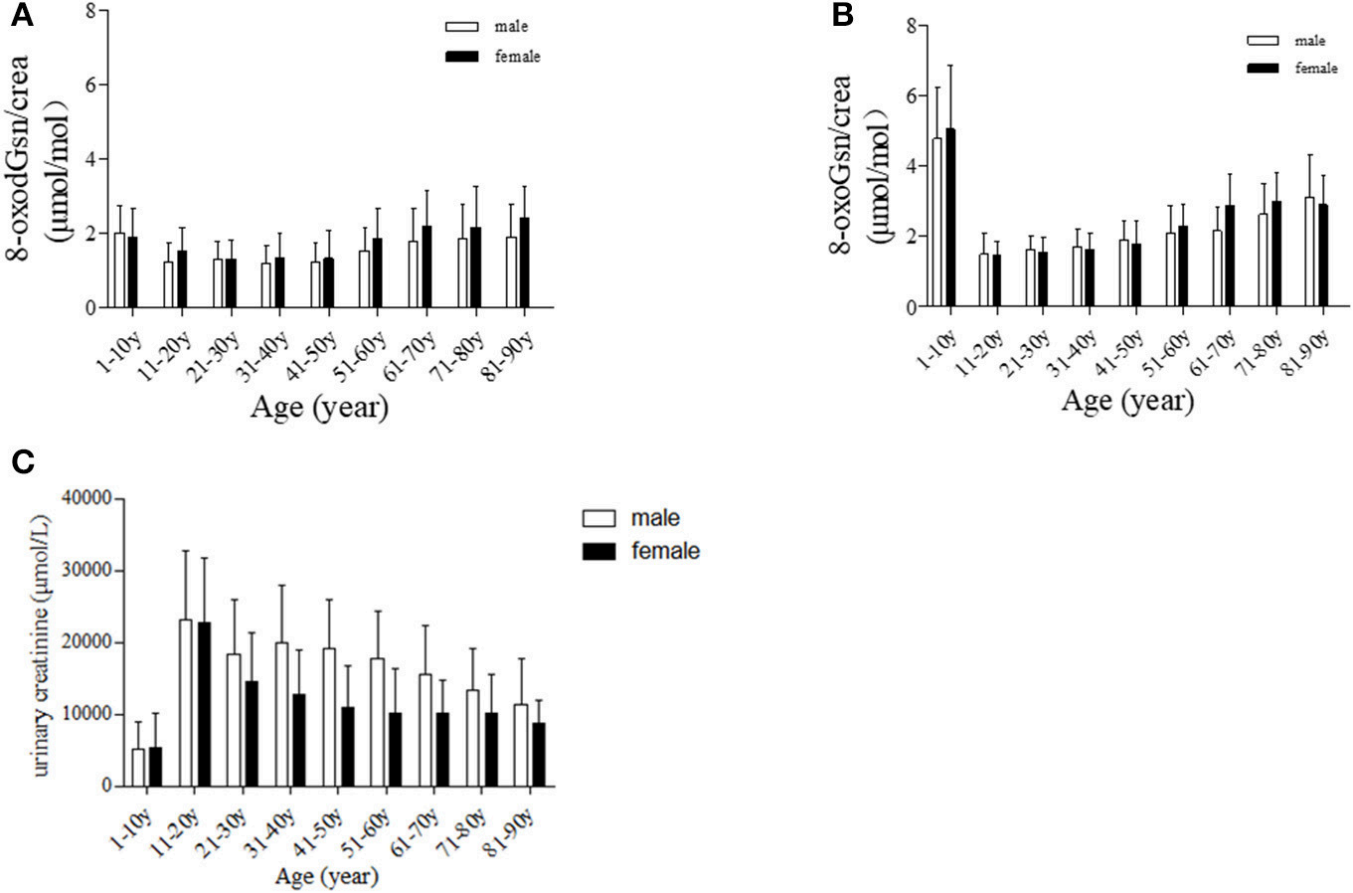
Age-dependent changes in urinary oxidized guanosine and creatinine. **(A)** Age-dependent changes in 8-oxodGsn. **(B)** Age-dependent changes in 8-oxoGsn. **(C)** Age-dependent changes in urinary creatinine.

We first compared the levels of oxidized guanosine in males and females after adjusting for age. The data are shown in Table 2 and Figure 2. There were no significant differences in these values between men and women under 60 years of age. However, in the subjects ≥61 years of age, the levels of 8-oxodGsn as well as 8-oxoGsn in the urine were much higher in females than in males. The content of oxidation products in females 51–60 years of age increased sharply, while the rate of increase in males remained slow. In males, the levels of 8-oxodGsn/crea in those 51–60 years of age was 1.2 times the value in those 41–50 years of age (1.53 ± 0.61 vs. 1.23 ± 0.50 μmol/mol), while in females, the ratio was 1.4 (1.88 ± 0.79 vs. 1.33 ± 0.74 μmol/mol). The ratio of 8-oxodGsn, 8-oxoGsn/crea increased 10% between those 41 and 50 years of age and those 51 and 60 years of age in males (from 1.90 ± 0.54 to 2.10 ± 0.77 μmol/mol), whereas the corresponding value for females reached as high as 30% (from 1.76 ± 0.67 to 2.30 ± 0.60 μmol/mol). The same tendencies were observed in those older than 61 years of age, although a small discrepancy was found in the 8-oxoGsn levels in those 81–90 years of age due to the small number of samples. The increase in oxidized guanosine and deoxyguanosine levels in females at later stages of life might be due to the decreased production of estrogen.

**Table 2:** Age- and gender-specific levels for urinary 8-oxodGsn/crea and 8-oxoGsn/crea (μmol/mol) derived from 1,228 normal subjects.

	**Age**								
	**1–10 years**	**11–20 years**	**21–30 years**	**31–40 years**	**41–50 years**	**51–60 years**	**61–70 years**	**71–80 years**	**81–90 years**
**8-oxodGsn**									
Male	2.01 ± 0.75	1.23 ± 0.52	1.30 ± 0.49	1.20 ± 0.48	1.23 ± 0.50	1.53 ± 0.61^*^^,^^a^^,^^b^	1.79 ± 0.88^*^^,^^a^^,^^b^	1.85 ± 0.92^a^	1.91 ± 0.89^a^
Female	1.91 ± 0.76	1.54 ± 0.62	1.31 ± 0.53	1.35 ± 0.67	1.33 ± 0.74	1.88 ± 0.79^b^	2.18 ± 0.96^a^^,^^b^	2.16 ± 1.10^a^	2.42 ± 0.83^a^
**8-oxoGsn**									
Male	4.76 ± 1.45	1.48 ± 0.61	1.62 ± 0.40	1.70 ± 0.50	1.90 ± 0.54^a^	2.1 ± 0.77^*^^,^^a^^,^^b^	2.15 ± 0.66^*^^,^^a^	2.62 ± 0.89^*^^,^^b^	3.12 ± 1.2^a^^,^^b^
Female	5.05 ± 1.79	1.44 ± 0.40	1.53 ± 0.44	1.60 ± 0.50	1.76 ± 0.67^b^	2.3 ± 0.60^b^	2.88 ± 0.88^a^^,^^b^	2.99 ± 0.80^a^	2.9 ± 0.81^a^

* *p < 0.05compared to age matched female group*.

a *p < 0.05 compared with gender matched 11–20 years group*.

b *p < 0.05 compared with the gender matched neighboring younger group*.

### Age-related changes in the contents of 8-oxodGsn and 8-oxoGsn in human urine

We previously showed that 8-oxodGsn and 8-oxoGsn accumulated in tissues and urine with the age-dependent progression of SAMR1 (Gan et al., 2012). In this population-based study, we found that the changes in two oxidized guanosine formats against aging were similar to those in SAMR1. 8-oxodGsn/crea started at 1.3 ± 0.49 μmol/mol in the 21- to 30-year-old group and increased gradually with aging and reached the maximum value of 2.07 ± 0.69 in the 81- to 90-year-old group. The same tendency was found in 8-oxoGsn/crea (μmol/mol): the concentration was 1.62 ± 0.40 μmol/mol in the 11- to 20-year-old group and sharply increased to 3.12 ± 1.20 μmol/mol in the 81- to 90-year-old group. We compared 8-oxodGsn/crea 8-oxoGsn/crea levels in each age group with the 11- to 20-year-old group, and also with the gender matched neighboring younger group. The results were shown in Table 2. The change in the pattern of oxidized guanosine may reflect degeneration of the anti-oxidation capacity in the elderly.

We next performed a Pearson correlation analysis to investigate the relationship between oxidized guanosine content and age. We set the correlation analysis to begin at 21 years of age and end at 90 years of age. The results from all 1,126 samples showed that the content of both 8-oxodGsn and 8-oxoGsn correlated positively with age (8-oxodGsn, *r* = 0.337, *p* < 0.001; 8-oxoGsn, *r* = 0.580, *p* < 0.001).

As the denominator, the content of creatinine plays a vital role in the calculation of the oxidized guanosine-to-creatinine ratio, any bias in the creatinine level can alter the results. We illustrated graph (C) over measured creatinine levels at the different ages in Figure 2. Participants 1–10 years of age exhibited relatively low levels of creatinine, while those 11–20 years of age exhibited higher levels. Creatinine correction may therefore not be suitable in these two groups. To avoid introducing errors, we didn't include these two age groups in the data analysis.

### Urinary content of 8-oxodGsn and 8-oxoGsn in infants and children

**Figure 3:**
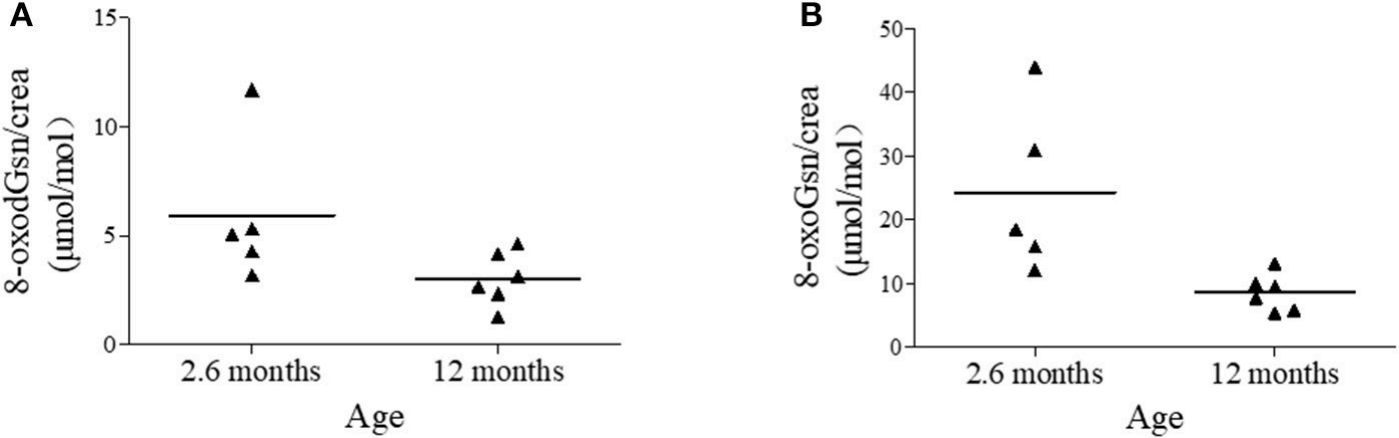
Urinary 8-oxodGsn and 8-oxoGsn levels in babies. **(A)** Urinary 8-oxodGsn in babies of average 2.6-months and 12 months. **(B)** Urinary 8-oxoGsn in babies of average 2.6-months and 12 months.

Interestingly, we found that children showed a much higher level of oxidized nucleic acids than older subjects, so we explored the oxidation state in babies. We selected 11 seemingly-healthy babies, 5 aged from 1 to 5 months (average age = 2.6 months) and 6 aged from 6 to 12 months, and measured the urinary 8-oxoguanosine. The findings for each infant are plotted in Figure 3. The 8-oxodGsn/crea (μmol/mol) values in the 6-month-old and 1-year-old babies were as high as 5.91 ± 3.34 and 3.03 ± 1.23 respectively, which are almost 3 and 1.5 times higher than the values in the participants 1–10 years of age, respectively. Markedly higher levels of 8-oxoGsn/crea (μmol/mol) were seen in 2.6-month-old and 1-year-old babies (24.25 ± 13.09 and 8.58 ± 2.90), being approximately 5- and 1.6-fold higher than in the 1- to 10-year-old group. There seems to be an imbalance between oxidation and anti-oxidation states in newborns.

## Discussion

Researchers have defined normal aging as a disease, a condition that can be “manipulated, treated, and delayed” (Nieuwenhuis-Mark, 2011), therefore, identifying the exact stage of aging process will be beneficial. A good aging marker should faithfully reflect the state of aging and not be influenced by other factors (Sprott, 2010), since the aging process itself is a risk factor for many diseases (Simm and Johnson, 2010).

Under the free radical theory of aging, urinary 8-oxodGsn and 8-oxoGsn are molecules that may reflect the oxidative state of the whole body rather than a specific organ, and these are promising biomarkers of aging. The European Standards Committee on Urinary (DNA) Lesion Analysis (ESCULA) was established to resolve the issue of inconsistency among methods. They concluded that MS- and EC-based assays were more reliable than ELISAs (Cooke et al., 2008). The first application of oxidized guanosine measurement based on HPLC-MS/MS was described by Weimann et al. (2002). Since then, many researchers have performed studies to improve the performance of the system. As a result, the UPLC-MS/MS method was established as the most robust among the previously reported methods in terms of the detection limit, detection time and accuracy. Our method is particularly suitable for use in routine measurement for large-scale epidemiological studies, since large numbers of urine samples can be analyzed in a reasonable duration (about 20 samples/h, 240 samples/day).

The influence of sex on the contents of urinary oxidized modified guanosine is controversial. Some studies have obtained lower values for females than for males (Andreoli et al., 2011), while others obtained conflicting results. We found that the excretion of 8-oxoGsn was not significantly different (*p* > 0.05) between males and premenopausal females; however, postmenopausal females excreted significantly more 8-oxoGsn than did males. This is similar to the results of Nakano et al. (2003), who investigated the 8-oxodGsn (not 8-oxoGsn) concentrations in 2507 healthy Japanese between 22 and 89 years of age. These results can be explained by the change in the levels of estrogen and iron. During menopause, a decrease in the production of estrogen might result in a reduced capacity for anti-oxidation. Iron is well known to cause oxidative stress by generating hydroxyl radicals via the Fenton reaction. Premenopausal females lose iron regularly by monthly menstruation, which reduces the urinary 8-oxodGsn excretion; as such, postmenopausal females have higher iron content and are prone to suffer from oxidative damage (Nakano et al., 2003).

We have taken a keen interest in the relationship between oxidation markers and age. Most previous studies have reported a rise in the urinary 8-oxodGsn level with age (Pilger et al., 2001; Sakano et al., 2009; Kaneko et al., 2012), while a few found no age-related difference (Nakano et al., 2003). Our previous study showed an age-dependent increase in the two biomarkers in mice, rats and monkeys (Gan et al., 2012; Shi et al., 2012; Nie et al., 2013). In the present study, the same trend was noted in humans. Interestingly, our result showed that humans excreted less oxidized guanosine than did animals. Compared with other studies, the current studies covered larger range of ages, from neonates to 90-year-olds. It is worthy of note that infants have extremely high urinary 8-oxodGsn/crea and 8-oxoGsn/crea levels. This may be because the process of childbirth is accompanied by an increase in oxidative aggression; the fetus moves from an intrauterine environment that is hypoxic to another with greater oxygen content. Alternatively, the antioxidant system of newborns is weak and may be insufficient to scavenge the excessive ROS. Along with the increase in age, the anti-oxidation systems develop quickly and mature. The lowest 8-oxodGsn and 8-oxoGsn levels appeared in the young adults (11–30 years of age). As people age, the antioxidant defense systems degenerate, and the levels of 8-oxodGsn and 8-oxoGsn increase gradually until the end of life. However, of note: although creatinine excretion is widely used as a method of normalizing the urinary excretion of analytes, the amount of creatinine produced each day is related to the muscle mass. Therefore, using creatinine to adjust the urinary concentration in those with extremely high (gymnasts) and low (babies) (Figure 2C) muscle contents will introduce great bias. Since there is no consistent evidence regarding the reference intervals of urinary 8-oxodGsn and 8-oxoGsn, it is hard to conclude whether the high levels of oxidized products in babies are a reflection of the real physical condition or the result of creatinine bias.

To date, most studies have dealt with urinary 8-oxodGsn, and a very limited number of studies have focused on 8-oxoGsn. This discrepancy in focus may be because 8-oxoGsn is an adduct from RNA, which does not pass on to the next generation. However, RNA plays an important role in protein translation, and any modification of its bases will directly lead to the formation of abnormal proteins. One important characteristic of aging is the accumulation of dysfunctional proteins. It is therefore reasonable to consider that 8-oxoGsn is a biomarker of aging.

Our study demonstrated that 8-oxoGsn is a better aging marker than 8-oxodGsn in two respects. First, the level of 8-oxoGsn was higher (approximately 2-fold) than 8-oxodGsn in age-matched counterparts. Second and more importantly, the levels of 8-oxoGsn correlated better with the rate of aging (Table 2 and Figure 1). The 8-oxoGsn content does not always correlate with chronological aging (Sprott, 2010) but instead reflects the actual physiological stage of aging.

In conclusion, a simple, rapid, sensitive and reliable methodology for the analysis of urinary excretion of 8-oxoGsn and 8-oxodGsn was developed. Based on this large population study, we concluded that 8-oxoGsn in urine may be a novel way to evaluate the aging process in adults.

## Ethics statement

This study was carried out in accordance with the recommendations of Guidelines for Biomedical Research Involving Human blood samples, the Institutional Ethics Committee of West China Hospital of Sichuan University, with written informed consent from all subjects. All subjects gave written informed consent in accordance with the Declaration of Helsinki. The protocol was approved by the Institutional Ethics Committee of West China Hospital of Sichuan University, the Number of the Approvement is 2014 Clinical Trial (NO.14).

## Author contributions

WG: design of the work; establish the UPLC MS method, the acquisition, analysis, or interpretation of data, drafting the work. X-LL: design of the work; the acquisition, analysis, or interpretation of data, drafting the work. TY: collect sample, measurement. Y-GZ: acquisition and analysis of data. T-TL and SW: collect sample. JD: measurement. L-LW: design of the work; final approval of the version to be published; and agreement to be accountable for all aspects of the work. J-PC: design of the work; final approval of the version to be published; revising it critically for important intellectual content; and agreement to be accountable for all aspects of the work.

### Conflict of interest statement

The authors declare that the research was conducted in the absence of any commercial or financial relationships that could be construed as a potential conflict of interest.
