# Supplementary material for: Urinary 8-oxo-7,8-dihydroguanosine as a Potential Biomarker of Aging
Source: Front Aging Neurosci. 2018 Feb 27;10:34. doi: 10.3389/fnagi.2018.00034 (PMC5835306; doi:10.3389/fnagi.2018.00034)
Supplement: Supplementary file 1 [file Table1.docx]

**Supplementary Table 1. UPLC conditions for the analytes**

| Analytes | Elute condition | Injection  volume | Run time |
| --- | --- | --- | --- |
| 8-oxo-dGuo &  8-oxoGuo | 0-1.8 min: 5% B, 0.3 ml/min; 1.8-2 min: 20% B, 0.3 ml/min; 2-3 min: 5% B, 0.3 ml/min | 2 μl | 3 min |

A: 0.1% formic acid

B: 100% methanol

**Supplementary Table 2.** basic conditions of physical health of the participants

|  | Mean ± SD | Reference Interval |
| --- | --- | --- |
| BMI (kg/m2) | 22.1±3.35 | 18.5-24.9 |
| SBP (mmHg) | 121.3±16.5 | 90-140 |
| DBP (mmHg) | 76.3±10.5 | 60-90 |
| FBG (mmol/L) | 5.16±0.33 | 3.9-5.9 |
| TG (mmol/L) | 1.09±0.38 | 0.29-1.83 |
| TC (mmol/L) | 4.73±0.58 | 2.80-5.70 |
| ALT (IU/L) | M:24.51±6.23 F: 20.33±7.13 | M:<50 F: <40 |
| AST (IU/L ) | M:25.75±4.81 F: 22.75±3.96 | M: <40 F: <35 |
| Cr (μmol/L) | M: 83.32±17.76 F: 78.37±15.11 | M:53-140 F:37-110 |
| BUN (mmol/L) | M: 6.24±1.27 F: 5.15±0.99 | M:3.20-8.86 F:2.77-8.75 |
| Current smoking | No | No |

BMI, body mass index; SBP, systolic blood pressure; DBP, diastolic blood pressure; FBG,fasting blood glucose; TG, triglyceride; TC, total cholesterol; Cr, creatinine; BUN, blood urea nitrogen.
